# Heterogeneity in pericyte inflammatory responses across age and species highlight the importance of human cell models

**DOI:** 10.1186/s13041-025-01209-7

**Published:** 2025-04-18

**Authors:** Taylor J. Stevenson, Kahee Lee, Susan Li, Johanna M. Montgomery, Kevin Y. Lee, Michael Dragunow

**Affiliations:** 1 Department of Pharmacology, University of Auckland, Auckland, New Zealand; 2 Centre for Brain Research, University of Auckland, Auckland, New Zealand; 3 Department of Physiology, University of Auckland, Auckland, New Zealand; 4 Department of Neurological Surgery, University of California San Francisco, San Francisco, United States of America

**Keywords:** Pericytes, Inflammation, Aging, Central nervous system

## Abstract

Pericytes in the central nervous system are essential for maintaining blood-brain barrier function, regulating blood flow, modulating immune responses, and interacting closely with surrounding cells of the neurovascular unit to support brain homeostasis. Increasing evidence has highlighted their involvement in age-related neuroinflammation, where their dysfunction may contribute to sustained inflammatory states associated with neurodegenerative disorders. Here, we compared inflammatory responses to lipopolysaccharide (LPS) in primary cerebral pericytes from neonatal and adult mice and adult humans. Our findings indicate that neonatal mouse pericytes display heightened inflammatory activation, with elevated levels of ICAM-1 and several cytokines, compared to adult mouse pericytes reflecting a more reactive phenotype. In contrast, adult mouse pericytes exhibited a significantly reduced cytokine release profile, suggesting lower responsiveness. Notably, while cytokine secretion patterns in adult human pericytes, in part, mirrored those in neonatal mouse pericytes, nitric oxide production, which was observed in mouse pericytes, was absent in the human cells. These results underscore species- and age-dependent variations in cellular behavior, emphasizing the importance of utilizing human brain cell systems when conducting research on neuroinflammation. Understanding these distinctions is vital for designing accurate studies and developing targeted therapies for neuroinflammatory conditions.

**Supplementary Information:**

The online version contains supplementary material available at 10.1186/s13041-025-01209-7.

Cerebral pericytes are important cells within the central nervous system (CNS), residing along the walls of capillaries. Here, they regulate key physiological functions including blood-brain barrier (BBB) integrity, cerebral blood flow, and maintenance of the extracellular environment. This provides structural and functional stability to the neurovascular unit [1]. Their location along the blood vessels uniquely positions them to directly interact with endothelial cells, neurons, and glial cells, allowing pericytes to serve as key communicators within the CNS microenvironment. These cells not only support the vascular barrier by controlling permeability but also play a vital role in immune surveillance, modulating immune cell trafficking, and mediating neuroinflammatory responses [1, 2]. As “gatekeepers” of the neurovascular unit, pericytes respond to inflammatory cues by releasing cytokines and chemokines, which can influence immune cell recruitment, making them central players in maintaining CNS homeostasis and neurovascular health [2, 3].

In recent years, pericytes have gained significant attention for their role in neuroinflammation and disease processes, particularly in aging and neurodegenerative diseases such as Alzheimer’s and Parkinson’s disease [1, 2, 4]. During aging, pericytes often exhibit a decline in function and structural integrity, which can impair their ability to maintain BBB stability and effectively regulate immune responses [5]. Dysfunctional pericytes contribute to a chronic state of low-grade inflammation, exacerbating the neuroinflammatory milieu that underlies many neurodegenerative conditions [6]. Their dysfunction and increased vulnerability in the aged brain may not only compromise BBB function but also amplify inflammatory signals, promoting a sustained inflammatory state that accelerates neurodegeneration [2–5]. The central role of pericytes in these processes positions them as early responders to neuroinflammatory triggers [7].

Much of our understanding of pericyte neuroinflammation comes from rodent models, yet species differences raise concerns about their relevance to human conditions. Neonatal mouse pericytes are commonly used in *in vitro* studies due to their accessibility, despite potential species- and age-dependent differences in inflammatory responses. This study aimed to compare the inflammatory responses of neonatal and adult mouse brain pericytes with adult human brain pericytes following bacterial endotoxin lipopolysaccharide (LPS; 5 or 40 ng/mL) [2] stimulation. Our results provide critical insights into species- and age-related differences to assess the suitability of rodent pericyte models and emphasize the need for more human-relevant models in neuroinflammation research.

Here, we cultured primary brain pericytes from wild-type neonatal and adult mice (C57BL/6J, postnatal day 3–7 and 7–12 months, respectively) and adult human tissue using established protocols (Additional file 1). To ensure consistency in culture conditions and facilitate direct comparison of inflammatory responses across species and ages, we used the same culturing protocol for both mouse and human brain pericytes (Additional file 1).

Our immunocytochemistry (ICC) results indicate that neonatal mouse pericytes display an exaggerated inflammatory state upon LPS exposure (24 h *in vitro*) when compared to mature adult mouse pericytes (Fig. 1A-G). This hyperactivity is reflected by the significantly elevated expression of intracellular adhesion molecule-1 (ICAM-1) (Fig. 1B, E). However, there were no statistically significant differences between neonatal and adult mouse pericytes for the expression of vascular adhesion molecule-1 (VCAM-1) (Fig. 1F) and monocyte chemoattractant protein-1 (MCP-1) (Fig. 1G). Elevated expression of ICAM-1 suggests that neonatal pericytes could adopt a more reactive phenotype, potentially driving greater immune cell recruitment and promoting an amplified inflammatory response within the CNS. Interestingly, neonatal and human brain pericytes showed similar levels of ICAM-1 and VCAM-1 expression (Fig. 1E, F), though primary human pericytes exhibited a significant increase in MCP-1 expression as assessed by ICC (Fig. 1G).

**Fig. 1:**
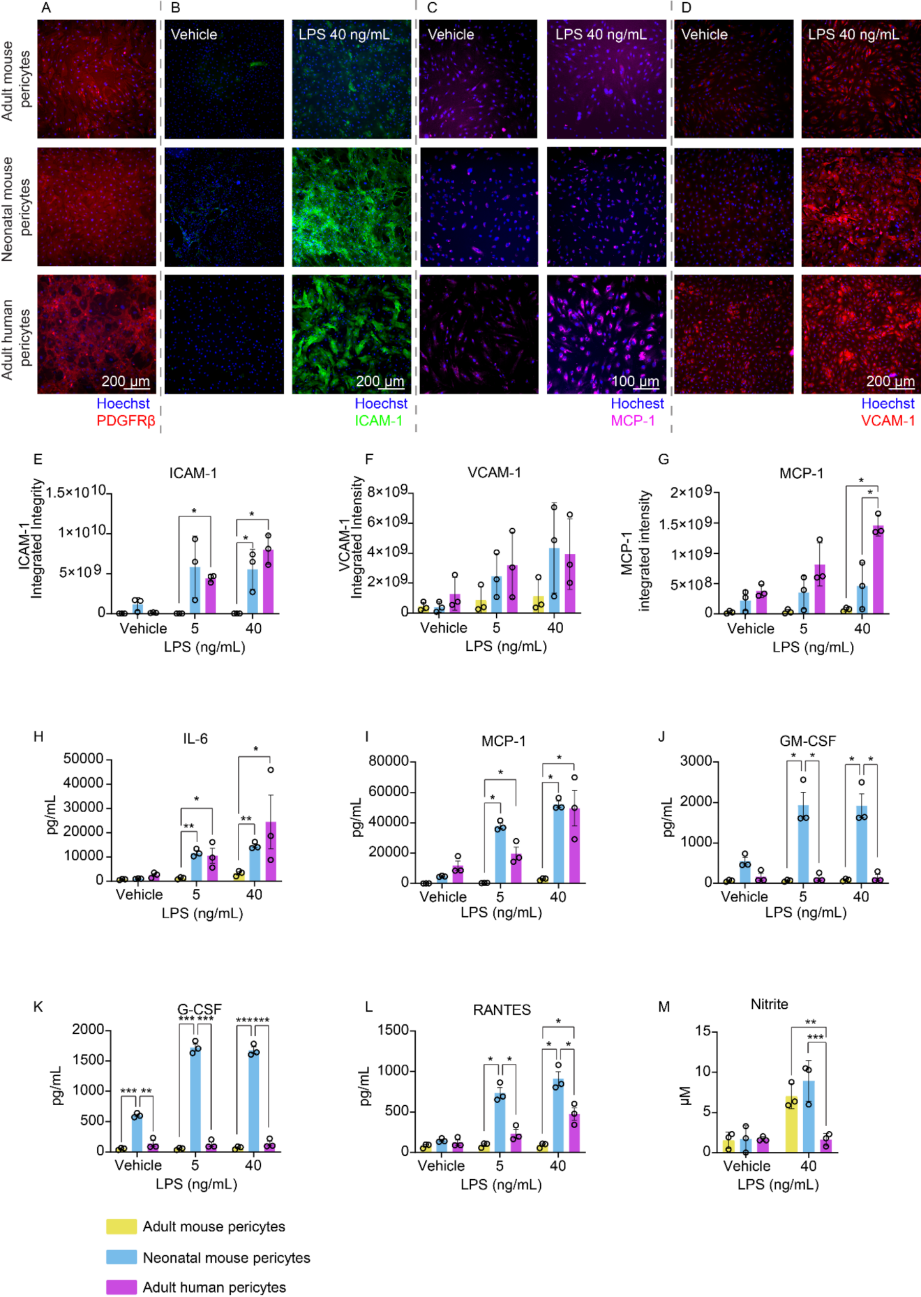
Neonatal mouse pericytes, adult mouse pericytes, and adult human pericytes display differential inflammatory responses. Representative images of primary adult mouse pericytes, primary neonatal mouse pericytes, and primary adult human brain pericytes displaying **A**) PDGFRβ expression, **B**) ICAM-1 expression after 24 h treatment with vehicle or LPS (40 ng/mL), **C**) MCP-1 expression after 24 h treatment with vehicle or LPS (40 ng/mL) and **D**) VCAM-1 expression after 24 h treatment with vehicle or LPS (40 ng/mL). Immunocytochemistry quantification of **E**) ICAM-1, **F**) VCAM-1, and **G**) MCP-1 expression after treatment with vehicle or LPS (40 ng/mL) in adult mouse pericytes, neonatal mouse pericytes and adult human pericytes. Quantification of cytokines and chemokines released into the media via cytometric bead array (CBA) for **H**) IL-6, **I**) MCP-1, **J**) GM-CSF, **K**) G-CSF and **L**) RANTES between vehicle and LPS (5 ng/mL or 40 ng/mL) in adult mouse pericytes, neonatal mouse pericytes, and adult human pericytes. **M**) Nitrite concentration quantified by Griess assay in adult mouse pericytes, neonatal mouse pericytes and adult human pericytes. Data was presented as the standard error of the mean from three independent culture sets (passages 5–7) from three independent biological replicates in all experiments. Data were statistically analyzed using Graphpad Prism 8 and a two-way ANOVA with Tukey’s multiple comparisons. *p < 0.05, **p < 0.01, ***p < 0.001

To further delineate age and species-specific inflammatory profiles, we analyzed cytokine and chemokine secretion patterns across neonatal mouse, adult mouse, and adult human pericytes via measuring their secretions in conditioned media 24 h post LPS treatment using a cytometric bead array (CBA) analysis (Additional file 1). Adult mouse pericytes demonstrated a markedly attenuated cytokine secretion profile, with significantly lower levels of most cytokines investigated, compared to both neonatal and human pericytes (Fig. 1H-L). Notably, neonatal mouse pericytes exhibited hypersecretion of granulocyte-macrophage colony-stimulating factor (GM-CSF), granulocyte colony stimulating factor (G-CSF) and regulated on activation normal T cell expressed and secreted (RANTES) (Fig. 1J-L) when compared to both human and adult mouse pericytes, highlighting an age-dependent elevation in the release of specific pro-inflammatory mediators. In contrast, human pericytes displayed cytokine release patterns that were comparable to neonatal mouse pericytes for IL-6 and MCP-1 (Fig. 1H-I), suggesting partial overlap in inflammatory response pathways despite species differences.

Lastly, we investigated nitric oxide (NO) release, a powerful pro-inflammatory agent. In mice, pericyte-derived NO can modulate BBB integrity, immune cell recruitment, and local oxidative stress, all of which are key factors in neurodegenerative disease pathology [8]. Both neonatal and adult mouse pericytes responded to LPS by releasing NO, as demonstrated by an increased nitrite concentration, an inert metabolite of NO oxidation and thus a proxy for NO production, via the Griess assay (Fig. 1M; Additional file 1). This was consistent with previous findings [8]. However, human pericytes did not release NO under similar conditions, underscoring a clear species-dependent difference in response to inflammatory stimuli.

Together, our data revealed age- and species-related differences in pericyte-mediated inflammation. Neonatal mouse pericytes displayed a hyper-inflammatory phenotype with elevated ICAM-1 expression and increased secretion of GM-CSF, G-CSF, and RANTES compared to adult pericytes. This aligns with studies showing heightened inflammatory activity observed in neonatal microglia [9] supporting the idea that the developing brain is highly vulnerable to infection and stress, which may lead to long-term CNS dysfunction [10]. In contrast, adult mouse pericytes demonstrated an attenuated inflammatory response, possibly due to immunosenescence, a process in which aging is associated with immunosuppressive activity, impairing the immune system’s ability to mount an efficient response to inflammatory stimuli while contributing to low-grade inflammation [11–13].

A key finding is the species-dependent difference in NO production. Unlike mouse pericytes, human pericytes did not produce NO in response to LPS. Previous studies suggest that species-specific regulation of nitric oxide synthase-2 (NOS2 or iNOS) in immune cells, such as macrophages, occurs via differential gene promoter activity and post-transcriptional mechanisms [14]. Whether pericytes exhibit similar regulatory differences remains unclear. Since endothelial cells produce NO via constitutive NOS3 (eNOS) activity, it is possible that human pericytes require endothelial-derived NO for signalling, rather than producing NO autonomously [15–17].

We acknowledge that the use of human pericytes from epilepsy patients, a condition associated with heightened neuroinflammation [18], is a limitation. Additionally, inherent age-related differences in human biopsy samples may have contributed to variability in our pericyte response. It is also possible that species- and age-dependent differences in pericyte physiology necessitate distinct *in vitro *requirements, which may have influenced the observed inflammatory outcomes. Moreover, we examined primary pericyte monocultures, though neuroinflammation involves multiple interacting cell types. The attenuated inflammatory response from adult mouse pericytes and the absence of NO production in human pericytes may stem from the lack of multi-cellular interactions or could represent an *in vitro *artifact, highlighting the need for *in vivo* validation or multi-cellular *in vitro* models.

Our findings highlight the need for strategic consideration of cell origin, age, and species in neuroinflammation research to ensure that comparative conclusions in *in vitro* systems are relevant and applicable to human conditions. Based on our data, human pericytes exhibit a more similar inflammatory profile to neonatal rather than adult mouse pericytes, particularly in terms of ICAM-1 expression and cytokine release patterns such as IL-6 and MCP-1. However, important species-specific differences remain, most notably the lack of NO production in human pericytes despite robust NO release from both neonatal and adult mouse pericytes. This underscores the limitations of relying solely on mouse models to study pericyte-mediated neuroinflammation, as key regulatory mechanisms may differ between species. While neonatal mouse pericytes may serve as a closer approximation of human cells than adult mouse pericytes, careful interpretation is required when extrapolating findings to human conditions.

## Electronic supplementary material

Below is the link to the electronic supplementary material.

Supplementary Material 1

## Data Availability

No datasets were generated or analyzed during the current study.

## Abbreviations

BBB: Blood Brain Barrier
CBA: Cytometric Bead Array
CNS: Central Nervous System
GM-CSF: Granulocyte Macrophage Colony-Stimulating Factor
G-CSF: Granulocyte Colony-Stimulating Factor
ICAM-1: Intercellular Adhesion Molecule-1
ICC: Immunocytochemistry
LPS: Lipopolysaccharide
MCP-1: Monocyte Chemoattractant Protein-1
PDGFRβ: Platelet Derived Growth Factor Receptor β
NO: Nitric Oxide
NOS: Nitric Oxide Synthase
RANTES: Regulated on Activation, Normal T Cell Expressed and Secreted
VCAM-1: Vascular Cell Adhesion Molecule-1
