# Supplementary material for: Heterogeneity in pericyte inflammatory responses across age and species highlight the importance of human cell models
Source: Mol Brain. 2025 Apr 18;18:37. doi: 10.1186/s13041-025-01209-7 (PMC12007128; doi:10.1186/s13041-025-01209-7)
Supplement: Supplementary file 1 — Supplementary Material 1 [file 13041_2025_1209_MOESM1_ESM.docx]

**Additional file 1**

**Materials and Methods**

**Mouse Brain Tissue culture**

For primary cell cultures, brain tissue was obtained from male or female C57BL/6 mice ranging between the ages of postnatal-day 3 – postnatal-day-7 (neonatal mice) and 7-12-months old (mature adult/middle-aged mice). All protocols used in this study were approved by the University of Auckland Animal Ethics Committee (ethics no. 2277) and procedures at the Vernon Janssen Unit (VJU) at the University of Auckland. All experiments and methods applied in this study were carried out within the approved guidelines.

Mouse *in vitro* cultures were adapted from previous studies^1–3^. Adult mice and neonatal mice were euthanized via carbon dioxide inhalation, followed by cervical dislocation and decapitation with large forceps. A sagittal cut beginning between the eyes from the posterior end to the anterior end of the head was made with a scalpel. Multiple cuts with small forceps were made in a half-circle around the top of the exposed skull and removed with tweezers. The entire brain was removed with a small scoop and gently rolled across a sterilized paper towel to remove the leptomeninges and external blood vessels. The whole brain was then placed in a sterilized petri dish containing 3 mL of chilled Hank’s balanced salt solution (HBSS; 14025-092; Invitrogen). Under a dissection microscope, the cerebellum, brain stem and olfactory bulbs were removed with fine tweezers. The brain was split into its’ two hemispheres wherein the midbrain and corpus callosum from each hemisphere were removed, leaving the entire forebrain intact. The forebrains were placed in a sterilized petri dish and ~2 mL HBSS (or enough volume to cover the forebrain) was gently poured over the forebrain. The forebrains were then minced with a scalpel for 5 minutes (or until very fine pieces were obtained). An enzymatic solution with HibernateA (A12475-01; Invitrogen), DNase-1 (18047-019; Invitrogen) and papain (LK003176; Worthington) was made up for tissue digestion (Supplementary Table 1). The minced tissue was washed down in the petri dish with 5 mL of the enzymatic solution with an electronic pipette, suctioned, and then placed into a 50 mL falcon tube. The falcon tube was placed in a rotator and left to digest for 15 minutes at 37°C, briefly triturated, and placed back in the rotator for another 15 minutes. 10 mL of complete media made up of DMEM/F12 (11330-057; Invitrogen) supplemented with GlutaMAX (35050061; Gibco) containing 10% fetal bovine serum (FBS; 8020; Moregate) and 1% Penicillin/Steptomycin (15140122; Gibco) was pipetted into the falcon tube to stop digestion. The entire volume within the falcon tube was passed through a 100 μm mesh cell strainer atop a separate 50 mL falcon tube and placed in a centrifuge at 1,000 RPM for 5 minutes. The supernatant was removed and the pellet was resuspended in 6 mL of complete media warmed to 37°C. The entire volume was then placed in a T25 flask at 37°C. Cells only >P5 from neonatal and adult mouse primary pericyte cultures were used to ensure 100% culture purity^3,4^.

Supplementary Table 1: Volumes used for enzymatic mix for mouse brain digestion.

| **Experimental group** | **HibernateA** | **DNase-1** | **Papain** |
| --- | --- | --- | --- |
| Adult mouse pericytes | 17.3 mL | 200 µL | 2.5 mL |
| Neonatal mouse pericytes | 8.65 mL | 100 µL | 1.25 mL |

**Human Brain Tissue**

Human brain tissue was obtained with informed consent from adult epileptic patients undergoing surgical resection at the Auckland City Hospital. All experiments and methods applied in this study were carried out within the approved guidelines from the University of Auckland Human Ethics Committee (ethics no. 3442). Human brain pericytes were isolated and maintained as previously described^1,3–5^. The cases used for human brain tissue in this study are provided in supplementary table 2. E208, E215, and E199 cases were used for ICC experiments, while E216, E219, and E213 cases were used for CBA and Griess assay.

Supplementary Table 2: Human biopsy cases used for primary human brain pericytes

| Case | Pathology | Region of resected specimen | Age (years) | Sex |
| --- | --- | --- | --- | --- |
| E215 | Epilepsy, Mesial temporal sclerosis (Grade 3) | Right anterior and posterior temporal lobe | 29 | F |
| E216 | Epilepsy, Mesial temporal sclerosis (Grade 3) | Left temporal lobe | 28 | M |
| E219 | Epilepsy, reduced hippocampal neuronal density | Right temporal lobe | 51 | F |
| E213 | Epilepsy, patchy gliosis, no cortical dysplasia, no neoplasia, presumed | Left anterior temporal lobe | 23 | M |
| E119 | Epilepsy, mesial temporal lobe damage | Right temporal lobe | 42 | M |
| E208 | Epilepsy, mesial temporal sclerosis | Left temporal lobe | 52 | F |

**Cell plating and passaging**

Cells were harvested for experiments by adding 2.5 mL 0.25% Trypsin, 1 mM ethylenediaminetetraacetic acid (EDTA; 25200056; Gibco) and incubated for 2–5 min at 37 °C to allow for cell detachment. Cells were then collected in warm DMEM:F12 media. 10 µL of 1:1 Trypan Blue (15-250-061; Gibco) and cell suspension was prepared and added to a hemocytometer for cell counting. Cells were re-suspended in the correct volume of DMEM:F12 to achieve a cell density of 5000 cells/well in a 96-well plate (Nunc). All plates were incubated for 3 days at 37 °C with 5% CO2 to allow for cell adherence before treatments were added.

**Lipopolysaccharide (LPS) treatment**

Neonatal and adult mouse pericytes and human pericyte were treated with LPS (Escherichia Coli O111:B4 (L2630; Sigma-Aldrich) once cells were fully adhered at either 5 ng/mL or 40 ng/mL. Once treated, pericytes were left for 24 hours before conditioned media was collected for a cytometric bead array (CBA) or fixed for immunocytochemistry.

**Immunocytochemistry**

Pericytes were fixed 24 hours post treatment with 50 μL of 4% paraformaldehyde (PFA) in PBS and left to fix for at least 10 minutes at room temperature (RT). PFA was aspirated from all wells and washed with 100 μL of PBS with 0.1% Triton X-100 (PBS-T) 3 x 5 minutes on a rocker. Primary antibodies were diluted in PBS containing 1% normal donkey serum and 30 μL pipetted into appropriate wells. The following primary antibodies were used in this study: anti-ICAM1 antibody (Abcam, ab53013; rabbit monoclonal, 1:250; RRID: AB_870702 ), anti-VCAM1 antibody (Abcam, ab134047; rabbit monoclonal, 1:250; RRID: AB_2721053), anti-MCP1 antibody (Abcam, ab25124; rabbit polyclonal, 1:250; RRID: AB_448636), and anti-PDGFRB antibody (R&D Systems, AF1042; goat polyclonal, 1:1000; RRID: AB_2162633). Primary antibodies were left to incubate overnight at 4°C on a rocker. The next day, 3 x 5 minute washing steps with PBS were subsequently performed. Alexa Fluor® dye-conjugated to secondary antibodies and Hoescht 33342 (#62249; Thermo ScientificTM) were diluted in PBS containing 1% normal donkey serum, and 30 μL pipetted into appropriate wells. Secondary antibodies were left to incubate at RT for 3 hours on a rocker, protected from light. Secondary antibodies used in this study include: donkey anti-rabbit Alexa Fluor-488 (Thermo Fisher Scientific, A-21206; 1:500; RRID: AB_2535792), and donkey anti-goat Alexa Fluor-488 (Thermo Fisher Scientific, A-11055; 1:500; RRID: AB_2534102). 3 x 5 minute washing steps with PBS were then performed.

**Imaging and analysis**

ImageXpress® Micro Confocal High-Content Imaging System (version 6.5.5; MetaXpress, Molecular Devices; 20x magnification objective lens; 0.9 NA) was utilized for imaging of plates after immunocytochemistry. The MetaXpress® High-content Image Acquisition and Analysis software (version 6.5.5.559; MetaXpress, Molecular Devices) was used for all image immunofluorescence analyses, measuring integrated intensity using the cell scoring analysis module.

**Cytometric bead array (CBA)**

Following LPS treatment, conditioned media was collected, centrifuged at 300 x g for 5 minutes and the supernatant frozen at – 20 °C. Frozen media from LPS treated plates were thawed at RT. Cytokine concentrations, including for RANTES (BD Biosciences, 558345, 558324), IL-6 (BD Biosciences, 558301, 558276), MCP-1 (BD Biosciences, 558342, 558287), G-CSF (BD Biosciences 560152, 558326), and GM-CSF (BD Biosciences, 558347, 558335), were measured using a cytometric bead array (CBA) (BS Biosciences, California, United States of America) as per the manufacturer’s instructions. CBA samples were run on an Acuri C6 flow cytometer (BC Biosciences). Analysis of data were carried out with FCAP-array software (version 3.0; BD Biosciences) to convert fluorescent intensity values to concentrations using an 11-point standard curve (0 – 100,000 pg/mL). CBA secretome data were normalized to pg/ml / 10,000 cells.

**Griess Assay**

Molecular Probes Greiss Reagent Kit for Nitrite determination (G-7921; Molecular Probes) was used. Following 40 ng/mL LPS treatment to induce maximal response, conditioned media was collected, centrifuged at 300 x g for 5 minutes and the supernatant collected. 20 μL of Greiss Reagent, 150 μL of the conditioned pericyte media and 130 μL of deionized water were mixed in a microplate. The mixture was incubated for 30 minutes at RT. A photometric reference sample was prepared by mixing 20 μL of Greiss Reagent and 280 mL of deionized water. The absorbance was measured at 548nm, relative to the reference sample in a spectrophotometric microplate reader. A standard curve of nitrite concentrations against absorbance readings was made, and nitrite concentrations corresponding to the absorbance of experimental samples from the standard plot were read to convert nitrite concentrations.

**Statistical analysis**

All experiments were performed in at least 3 independent biological replicates. Data were presented as +/- standard error of the mean from at least 3 independent cases in all experiments. Data visualization and appropriate statistical analyses (two-way ANOVA and T-test of significance) were performed using GraphPad Prism® (version 9.3.1). Normality was assessed using the Shapiro-Wilk test, with normality considered satisfied if alpha > 0.05. All datasets passed the normality test. In tests that detected significance, post-hoc analyses (Tukey’s multiple comparisons test) were performed if required. Statistical significance was set as p < 0.05.

**Results**

Supplementary Table 3: Summary of responses to LPS between adult mouse pericytes, neonatal mouse pericytes and adult human pericytes

| Cell type | Responses to LPS | | | | | | | | |
| --- | --- | --- | --- | --- | --- | --- | --- | --- | --- |
|  | ICC | | | Cellular secretions | | | | | |
|  | ICAM-1 | VCAM-1 | MCP-1 | IL-6 | MCP-1 | GM-CSF | G-CSF | RANTES | NO |
| Adult mouse pericyte | ↑ | ↑ | ↑ | ↑ | ↑ | ↑ | ↑ | ↑ | ↑↑↑ |
| Neonatal mouse pericyte | ↑ ↑↑ | ↑ ↑↑ | ↑ ↑ | ↑↑↑ | ↑↑↑ | ↑↑↑ | ↑↑↑ | ↑↑↑ | ↑↑↑ |
| Adult human pericytes | ↑ ↑↑ | ↑ ↑↑ | ↑ ↑↑ | ↑↑↑ | ↑↑↑ | ↑ | ↑ | ↑↑ | - |

**References**

1. Park, T. I.-H., Smyth, L. C. D., Aalderink, M., Woolf, Z. R., Rustenhoven, J., Lee, K., Jansson, D., Smith, A., Feng, S., Correia, J., Heppner, P., Schweder, P., Mee, E. & Dragunow, M. Routine culture and study of adult human brain cells from neurosurgical specimens. *Nat Protoc* **17**, 190–221 (2022).

2. Woolf, Z., Stevenson, T. J., Lee, K., Jung, Y., Park, T. I. H., Curtis, M. A., Montgomery, J. M. & Dragunow, M. Isolation of adult mouse microglia using their in vitro adherent properties. *STAR Protocols* **2**, 100518 (2021).

3. Jansson, D., Rustenhoven, J., Feng, S., Hurley, D., Oldfield, R. L., Bergin, P. S., Mee, E. W., Faull, R. L. & Dragunow, M. A role for human brain pericytes in neuroinflammation. *J Neuroinflammation* **11**, 104 (2014).

4. Smyth, L. C. D., Rustenhoven, J., Park, T. I.-H., Schweder, P., Jansson, D., Heppner, P. A., O’Carroll, S. J., Mee, E. W., Faull, R. L. M., Curtis, M. & Dragunow, M. Unique and shared inflammatory profiles of human brain endothelia and pericytes. *J Neuroinflammation* **15**, 138 (2018).

5. Stevenson, T. J., Johnson, R. H., Savistchenko, J., Rustenhoven, J., Woolf, Z., Smyth, L. C. D., Murray, H. C., Faull, R. L. M., Correia, J., Schweder, P., Heppner, P., Turner, C., Melki, R., Dieriks, B. V., Curtis, M. A. & Dragunow, M. Pericytes take up and degrade α-synuclein but succumb to apoptosis under cellular stress. *Sci Rep* **12**, 17314 (2022).
